# Machine learning prediction of the adverse outcome for nontraumatic subarachnoid hemorrhage patients

**DOI:** 10.1002/acn3.51208

**Published:** 2020-09-29

**Authors:** Duo Yu, George W. Williams, David Aguilar, José‐Miguel Yamal, Vahed Maroufy, Xueying Wang, Chenguang Zhang, Yuefan Huang, Yuxuan Gu, Yashar Talebi, Hulin Wu

**Affiliations:** ^1^ Department of Biostatistics & Data Science School of Public Health The University of Texas Health Science Center at Houston (UTHealth) Houston Texas USA; ^2^ Department of Anesthesiology McGovern Medical School The University of Texas Health Science Center at Houston (UTHealth) Houston Texas USA; ^3^ Department of Medicine McGovern Medical School The University of Texas Health Science Center at Houston (UTHealth) Houston Texas USA; ^4^ Department of Epidemiology, Human Genetics, and Environmental Sciences School of Public Health University of Texas Health Science Center at Houston Houston Texas USA

## Abstract

**Objective:**

Subarachnoid hemorrhage (SAH) is often devastating with increased early mortality, particularly in those with presumed delayed cerebral ischemia (DCI). The ability to accurately predict survival for SAH patients during the hospital course would provide valuable information for healthcare providers, patients, and families. This study aims to utilize electronic health record (EHR) data and machine learning approaches to predict the adverse outcome for nontraumatic SAH adult patients.

**Methods:**

The cohort included nontraumatic SAH patients treated with vasopressors for presumed DCI from a large EHR database, the Cerner Health Facts^®^ EMR database (2000–2014). The outcome of interest was the adverse outcome, defined as death in hospital or discharged to hospice. Machine learning‐based models were developed and primarily assessed by area under the receiver operating characteristic curve (AUC).

**Results:**

A total of 2467 nontraumatic SAH patients (64% female; median age [interquartile range]: 56 [47–66]) who were treated with vasopressors for presumed DCI were included in the study. 934 (38%) patients died or were discharged to hospice. The model achieved an AUC of 0.88 (95% CI, 0.84–0.92) with only the initial 24 h EHR data, and 0.94 (95% CI, 0.92–0.96) after the next 24 h.

**Interpretation:**

EHR data and machine learning models can accurately predict the risk of the adverse outcome for critically ill nontraumatic SAH patients. It is possible to use EHR data and machine learning techniques to help with clinical decision‐making.

## Introduction

Subarachnoid hemorrhage (SAH) is a life‐threatening stroke that commonly affects individuals in midlife and often results in a substantial loss of productive life years among survivors.^1^, ^2^ Nontraumatic SAH is commonly caused by rupture of an intracranial aneurysm.^2^ While hospitalization for aneurysmal SAH is relatively uncommon, with an estimated rate of 14.5 per 100,000 U.S,^3^ it represents a potentially devastating condition with subsequent disability or even death. The reported in‐hospital mortality ranges from 25 to 50%.^2^ A particularly severe complication following SAH is delayed cerebral ischemia (DCI) and the development of DCI results in even higher mortality and disability. In this higher risk cohort where vasopressor treatment is used to induce hypertension for presumed DCI,^2^, ^4^, ^5^ it would be helpful to accurately predict subsequent outcomes. Such information could assist clinicians in decision making and provide prognostic information for patients and families to inform further decisions.

The availability of large and diverse clinical data from Electronic Health Record (EHR) has the potential to deliver evidence‐based and personalized medicine. The richness of historical clinical information might be predictive for future diseases and outcomes of interest. Modern statistical and machine learning predictive models could also assist clinicians in clinical decision making, since they are capable of utilizing multiple sources of data, and identifying complex patterns not recognized by traditional statistical techniques. Many predictive models have been developed based on EHR for varying clinical outcomes, such as in‐hospital mortality and readmission.^6^, ^7^, ^8^, ^9^ However, most current clinical predictive models for SAH are rarely used in practice, partially due to the limitations in generalizability and predictive performance. These weaknesses of current models are mainly due to small derivation cohorts, lack of validation, difficulties with missing data, and limited ease of use.^10^ In addition, very few studies examined mortality as the outcome. In one analysis that examined mortality, the discrimination was low (the area under the receiver operating characteristic curve [AUC] is 0.76), which limits its usefulness in clinical practice.^11^ Therefore, a more reliable model to predict the risk of mortality in critically ill SAH patients is needed.

This study aims to use a large EHR database to predict the risk of the adverse outcome for nontraumatic SAH patients treated with induced hypertension. To the best of our knowledge, there has been no attempt to predict the risk of the adverse outcome for nontraumatic SAH patients using machine learning approaches based on EHR data.

## Methods

### Data sources

Data for this study were extracted from the Cerner Health Facts^®^ EMR database, which comprises de‐identified EHR data from over 700 hospitals and clinics in the United States. Cerner Health Facts^®^ EHR database includes structured data such as patient demographics, diagnoses, procedures, lab results, medications, vital signs, and other clinical observations. We utilized EHR data that were collected between 2000 and 2014. This study was approved by our local institutional review board (IRB). We followed the “Guidelines for Developing and Reporting Machine Learning Predictive Models in Biomedical Research: A Multidisciplinary View.”^12^

### Identification of the cohort with nontraumatic sah and primary outcome

We included patients who were diagnosed with SAH based on the *International Classification of Diseases, Ninth Revision, Clinical Modification* (ICD‐9‐CM) diagnosis code ICD‐9‐CM 430 who were treated with induced hypertension with vasopressors (norepinephrine, phenylephrine, and dopamine). To avoid the potential confounding effect of trauma, we excluded patients diagnosed with traumatic SAH (ICD‐9‐CM codes 800.0–804.9, 850.0–854.1, and 873.0–873.9). We also excluded patients age less than 17 years. The primary outcome was the adverse outcome, defined as death in hospital or discharged to hospice.

### Prediction setting and machine learning methods

**Figure 1:**
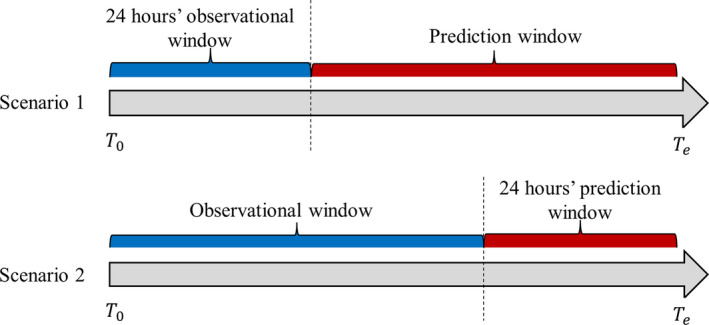
Relation of the observational window and prediction window in the two prediction scenarios. *T*_0_ and *T_e_* denoted the visit start and end time.

The primary objective of this study was to predict the risk of the adverse outcome for nontraumatic SAH patients who were treated with vasopressors in two scenarios (Fig. 1). In Scenario 1, we aimed to predict the risk of the adverse outcome using information based on the initial specified period’s EHR data after hospital admission (24, 48, and 72 h EHR data). In Scenario 2, we aimed to predict the risk of the adverse outcome using information from admission up to the last specified period’s hospitalization (24, 48, and 72 h before discharge). The potential predictors in this study were baseline demographic variables (age, gender, race, and marital status), categorical vasopressor treatment (dopamine, norepinephrine, and phenylephrine), categorical procedure codes, binary medication and diagnosis variables, and numerical results from lab tests, vital signs, and clinical observations. The missing data from lab tests, vital signs, and clinical observations were imputed with MissForest.^13^

We explored various machine learning methods such as the elastic net regularized logistic regression approach, support vector machine (SVM), random forest, gradient boosting machine (GBM), XGBoost, and multilayer perceptron (MLP). We mainly report the results from the Elastic Net method (implemented with R package caret^14^) due to its good interpretability and prediction accuracy. The cohort was firstly randomly split into training (70%) and validation (30%) data sets. Then, we derived sub‐cohorts for model development and evaluation in different prediction scenarios. Therefore, the training and validation datasets in the following prediction were subsets of original training and validation datasets. The AUC and its corresponding 95% CI for each of the machine learning prediction models were reported based on the validation data sets. The detailed variable description, leakage detection, and model validation measures can be found in the Supplementary Materials.

## Results

The final cohort size that met the inclusion and exclusion criterion was 2467, in which 934 experienced the outcome events (38%). The median age was 56 years (interquartile range, 47–66), and the majority of patients were female (65%). The median length of stay was 14 days (Table 1). The Glasgow Coma Scale (GCS) was available for 995 patients (40%) in this cohort, and the average value was 11 (Table 1). A total of 83 labs and clinical observation variables had missing values with an average magnitude of 46%. The detailed missing rates summary can be found in Table S1.

**Table 1:** Baseline characteristics of patients.

Characteristics	Model development cohort, n = 1747	Validation cohort, n = 720
Mortality (%)	676 (39)	258 (36)
Female sex (%)	1129 (65)	459 (64)
Median LOS in days (IQR)	14 (5–23)	15 (6–24)
Median age in years (IQR)	56 (47–66)	56 (46–66)
Age		
<40 y (%)	214 (12)	76 (11)
40–49 y (%)	310 (18)	157 (22)
50–59 y (%)	506 (29)	197 (27)
60–69 y (%)	388 (22)	159 (22)
>70 y (%)	329 (19)	131 (18)
Race		
White (%)	1175 (67)	473 (66)
African American (%)	380 (22)	177 (25)
Others (%)	192 (11)	70 (9)
Marital status		
Single (%)	444 (25)	196 (27)
Married (%)	812 (46)	323 (45)
Divorced (%)	181 (10)	85 (12)
Widowed (%)	134 (8)	44 (6)
Unknown (%)	176 (10)	72 (10)
First vasopressor		
Phenylephrine (%)	871 (50)	358 (50)
Norepinephrine (%)	497 (28)	206 (28)
Dopamine (%)	379 (22)	156 (22)
GCS^1^ (*n* = 995)		
3–5 (%)	152 (23)	43 (16)
6–8 (%)	83 (12)	33 (13)
9–11 (%)	74 (11)	39 (15)
12–14 (%)	122 (18)	40 (15)
15 (%)	236 (35)	106 (41)

LOS, hospital length of stay; GCS, Glasgow Coma Scale.

^1^ The GCS was available and analyzed for 995 (40%) patients.

In Scenario 1, where the length of the observational window was fixed to 24, 48, or 72 h, the sample size of each cohort was 816, 1139, and 1184, respectively. The differences in sample size were driven by the requirement that each patient have a long enough period of EHR data (for example, 24 h for the fixed 24 h observational window case) and have vasopressor assigned during the observational window. The AUC of predicting the risk of the adverse outcome after 24 h hospitalization was 0.88 (95% confidence interval [CI] = [0.84–0.92]), (Table 2). As the length of hospitalization increases to 48 and 72 h, the AUC was slightly lower: 48‐h AUC 0.86 (95% CI 0.82–0.89) and 72‐h AUC 0.84 (95% CI 0.79–0.88). In Scenario 2, where the length of the predictive window was fixed, the sample size was 2153, 1941, and 1821 in each case. The AUC of predicting the risk of the adverse outcome after the next 24 h hospitalization was 0.94 (95% CI = [0.92–0.96]). The AUCs slightly decreased with the increase in the length of the prediction window. The AUCs for predicting the adverse outcome after the next 48‐h and 72 h were 0.93 (95% CI 0.90–0.95) and 0.91 (95% CI 0.89–0.94), respectively.

**Table 2:** Prediction accuracy summary for Scenario 1 and 2 by using the elastic net regularized logistic regression model. The Scenario 1 prediction used the EHR data from first 24, 48, and 72 h after hospital admission; the Scenario 2 prediction used the EHR data up until 24, 48, and 72 h prior to hospital discharge or death.

Scenario 1	Sample size	Predictive window (days)	AUC, 95% CI
		Median (IQR)	
Predict with first 24 h	816	8.70 (1.63–18.57)	0.88, [0.84, 0.92]
Predict with first 48 h	1139	11.78 (3.67–20.45)	0.86, [0.82, 0.89]
Predict with first 72 h	1184	12.58 (5.67–20.60)	0.84, [0.79, 0.88]

Scenario 2	Sample size	Observational window (days)	AUC, 95% CI
		Median (IQR)	
Predict for next 24 h	2153	15.59 (7.78–23.91)	0.94, [0.92, 0.96]
Predict for next 48 h	1941	15.85 (9.38–24.36)	0.93, [0.90, 0.95]
Predict for next 72 h	1821	15.77 (9.65–23.99)	0.91, [0.89, 0.94]

Other model evaluation criteria, such as sensitivity, specificity, positive predictive value, and negative predictive value (Table S2). All of the elastic net penalized logistic regression models fit the data well (the Hosmer–Lemeshow test *P*‐values >0.05, Table S3). Other machine learning methods implemented using different Auto Machine Learning (Auto‐ML) software platforms confirmed the results from the elastic net logistic regression models (Table S4).

**Figure 2:**
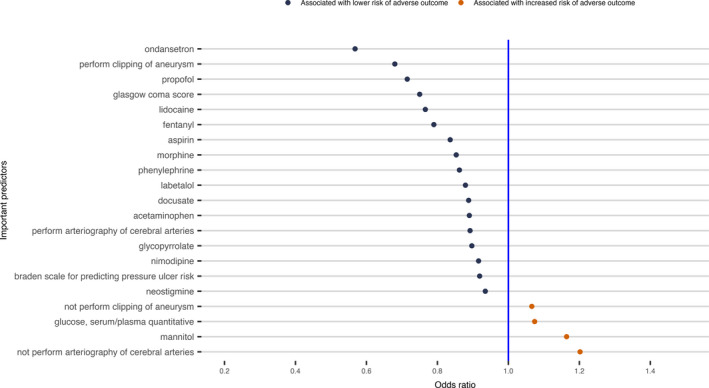
Variables and corresponding odds ratios in the elastic net regularized logistic regression model for predicting the risk of the adverse outcome with the first 24 h EHR data for nontraumatic SAH adult patients. Clipping of aneurysm is categorized as three levels: perform clipping, not perform clipping but performed other procedures, none of the procedures is performed based on the EHR data. In the final predictive model, perform clipping, and not perform clipping but performed other procedures are included.

**Figure 3:**
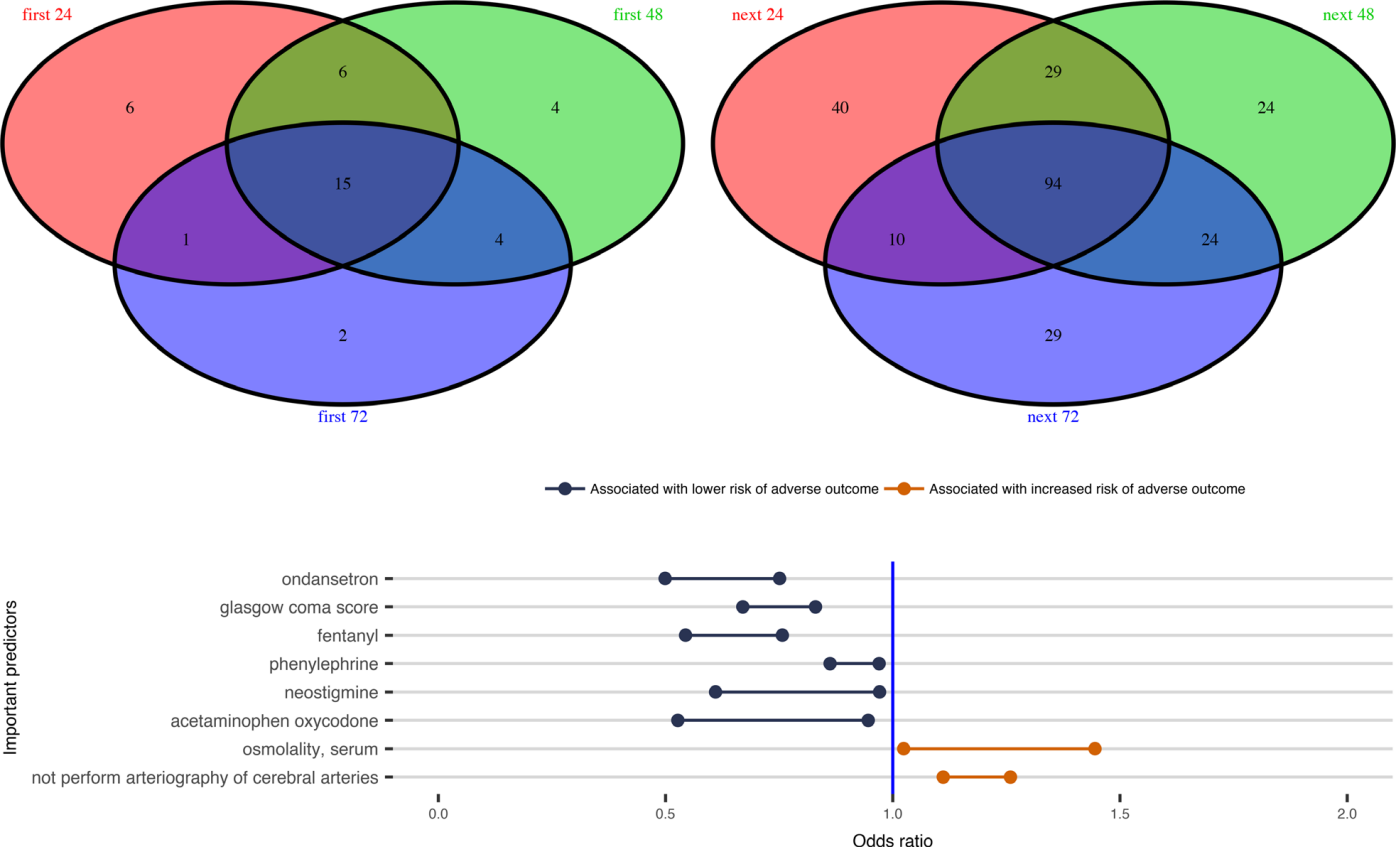
Top two panels are Venn diagrams for variables in prediction Scenario 1(left) and Scenario 2 (right). A total of 15 and 94 predictors were commonly included in the final predictive models in Scenario 1 and 2, respectively. Their detailed predictor names and corresponding odds ratios were shown in supplemental materials (Fig. S6–S7). The bottom panel is the odds ratios of 8 predictors that were included in all six prediction models for the two scenarios.

Those variables that were included in the final elastic net regularized logistic regression model were considered to be informative for predicting the risk of the adverse outcome. The prediction model included 26 of 185 variables using the data of the first 24 h EHR in Scenario 1 (Fig. 2). For the case in Scenario 2 to predict death using the data up to the last 24 h EHR, the final model selected 171 of 359 predictors (Fig. S1). Fifteen variables were included in all the prediction models for the three cases in Scenario 1(Fig. S6), while 94 variables were included in all the prediction models for the three cases in Scenario 2 (Fig. S7). There were 8 variables included in all six predictive models for the two Scenarios (Fig. 3).

## Discussion

While EHR systems are generally adopted in hospitals and clinics, to the best of our knowledge, there has been no attempt to predict the risk of the adverse outcome for nontraumatic SAH patients using machine learning approaches based on EHR data. In this study, we developed predictive models to predict the adverse outcome, defined as the in‐hospital mortality and discharge to hospice for nontraumatic SAH patient using the Cerner Health Facts^®^ database. The logistic regression models with elastic net penalty and other machine learning predictive models were able to accurately predict the adverse outcome in two scenarios: predicting the risk of the adverse outcome after first 24–72 h hospital admission and predicting the risk of the adverse outcome after next 24–72 h hospitalization. In Scenario 1, we used the early data (the first 24–72 h); in Scenario 2, we used all hospital information until the last 24–72 h before discharge or death. Both scenarios yielded predictive models with higher accuracy compared to the most recent mortality predictions with AUC of 0.76 (95% CI, 0.69–0.82) using traditional methods with data derived from clinical trials and observational clinical data.^11^

When comparing the performance of our predictive models from Scenario 1 and Scenario 2, we observed that the prediction accuracy of Scenario 2 was higher than that in Scenario 1 (Table 2). The AUC in Scenario 2 ranged from 0.91 to 0.94, whereas it ranged from 0.84 to 0.88 in Scenario 1. The difference in prediction accuracy between the two scenarios may be related to the larger sample size, longer observational window and shorter predictive window of Scenario 2 than Scenario 1. Within each scenario, we also identified slight differences in the prediction model performance as the observation and prediction windows varied. In Scenario 1, a decreasing trend of prediction accuracy was seen when the observed length of hospitalization duration increased from 24 to 72 h. This slightly decreasing trend may be due to the increased length of the predictive window, as the median length of the predictive window increased from 8.7 to 12.6 days. In Scenario 2, the models were most accurate at predicting within a shorter time period, with the highest AUC for the next 24 h and slightly decreasing in accuracy to predict the adverse outcome within the next 48‐h and 72 h. The decreasing sample size as the prediction window increased may have also contributed to the decreased trend of predictive accuracy in Scenario 2.

Utilizing machine learning methods, we identified several variables that were associated with the adverse outcome following nontraumatic SAH. Given the observational nature of the study, we are unable to determine the exact causal relationship between these predictive variables and the adverse outcome. Some predictors could be directly involved in the pathophysiology leading to increased probability of the adverse outcome, while other variables may be markers, some unexpected, for impending adverse outcome. Particularly, we identified 26 clinical variables for predicting the risk of the adverse outcome using the first 24 h EHR data (Fig. 2), including clipping of aneurysm and arteriography of cerebral arteries, which are two important treatments for SAH patients and would be expected to improve the outcomes. Cefazolin and aspirin were also associated with a decreased risk of the adverse outcome, since they are often used for patients who had invasive procedures, such as clipping and arteriography of cerebral arteries procedures.^15^ We confirmed that the medication, nimodipine, which is the only proved effective treatment for preventing DCI, is associated with improved outcomes.^16^. We also observed that labetalol was associated with a decreased risk of the adverse outcome, this is presumably because it is often used to avoid increase in blood pressure that might cause aneurysm rupture for aneurysmal SAH patients.^17^ Other variables such as the numerical Glasgow coma score (GCS) (range from 1 to 15), Braden scale for predicting pressure ulcer risk, and the use of mannitol and glucose may be reflective of SAH severity, and thus, associated with increased probability of the adverse outcome. Particularly, treatment with glucose would most likely be required in cases of severe hypoglycemia, which has been a risk marker of increased probability of the adverse outcome for critically ill patients.^18^ Laboratory variables such as glucose (hyperglycemia), anion gap acidosis, renal function, and serum osmolality would also be expected to be associated with adverse clinical outcomes.^19^ Finally, our recent work has demonstrated a beneficial association between phenylephrine use and the adverse outcome in this population.^20^ Our machine learning predictive models from all six cases (Fig. 3 bottom panel) further confirmed that the phenylephrine use could associate with a reduced probability of adverse outcome after adjusting many other confounding factors in the predictive models.

Other variables informative in predicting the adverse outcome were less expected. For example, certain medications used to treat symptoms such as pain (acetaminophen‐hydrocodone, fentanyl, acetaminophen and morphine), anxiety (propofol), nausea/vomiting (ondansetron), and constipation (docusate) were associated with improved outcomes (Fig. 2). While these variables may not be reflective of the pathophysiology of SAH, they are likely indicators of neurologic status following SAH, as a patient with very severe neurologic injury may not be able to manifest pain or other symptoms. Other medications such as those used for anesthesia procedures, for example, propofol, lidocaine, glycopyrrolate, and neostigmine, may also be reflective of procedures that require general anesthesia that, in turn, may be associated with improved outcomes. Our result also suggests that the heavier weight, strongly correlated with BMI (*r* = 0.83, data not shown), was associated with reduced probability of the adverse outcome, which is difficult to explain. But this so‐called “obesity paradox” was also observed in a recent nontraumatic SAH study by Elliot et al.’s.^21^

Despite the strengths of machine learning methods, there are several limitations that should be acknowledged to our methods and complexity of EHR data. We only used the structured EHR data to develop the prediction models since we were not able to access the unstructured EHR data such as clinical notes, CT scan and other imaging data due to difficulties to de‐identify these unstructured EHR data for patient privacy protection. Thus, the derived variables and other potential predictors related to SAH adverse outcome such as WFNS, aneurysm size, and Hunt and Hess grade, and Fisher grade were not used in our prediction models. Nonetheless, our prediction models achieved a higher prediction accuracy only using the raw structured EHR data than those using these well‐designed and tailored clinical variables or predictors for SAH patients.^11^ Also, since the EHR system has been predominantly designed to collect the data to support clinical practice, documentation and billing purpose, these data might have sampling bias and include high missing rates for different clinical variables. We explored different missing data imputation strategies and similar conclusions were achieved using different missing data methods (the results not shown due to space limitation). Ideally, further validation studies using different databases or well‐designed clinical studies are warranted to confirm our findings based on machine learning predictive models. Finally, we have focused on the binary adverse outcome prediction for nontraumatic SAH patients with vasopressor treatments. Future studies should be performed to generalize our prediction models to more general SAH patients with other clinical outcomes, including time‐to‐death outcome for survival models.

Our findings suggest that machine learning models can achieve high accuracy for predicting the adverse outcome for nontraumatic SAH patient using raw EHR data. The EHR‐based prediction model is more accurate than traditional models using the a priori selected clinical variables and predictors. In the clinical practice, this predictive model can serve as another source of agnostic assessment that is independent of practitioner experience and provide additional assurance to families when considering ongoing intervention.

## Conflict of Interest

Nothing to report.

## Supporting information


**Table S1.** Missing rate summary.
**Table S2.** Model discrimination summary.
**Table S3.** Model calibration summary.
**Table S4.** Auto machine learning implement comparison.
**Figure S1.** Important variables for prediction with first 48 h EHR data.
**Figure S2.** Important variables for prediction with first 48 h EHR data.
**Figure S3.** Important variables for prediction after the next 24 h.
**Figure S4.** Important variables for prediction after the next 48 h.
**Figure S5.** Important variables for prediction after the next 72 h.
**Figure S6.** Variables commonly included with first 24, 48, 72 h EHR data.
**Figure S7.** Variables commonly included for the prediction after the next 24, 48, 72 h.

## References

[1] Macdonald RL , Schweizer TA . Spontaneous subarachnoid haemorrhage. The Lancet 2017;389:655–666. 10.1016/S0140-6736(16)30668-7 27637674

[2] Lawton MT , Vates GE . Subarachnoid hemorrhage. N Engl J Med 2017;377:257–266. 28723321 10.1056/NEJMcp1605827

[3] Shea AM , Reed SD , Curtis LH , et al. Characteristics of nontraumatic subarachnoid hemorrhage in the United States in 2003. Neurosurgery 2007;61:1131–1138. 18162891 10.1227/01.neu.0000306090.30517.ae

[4] Connolly ES Jr , Rabinstein AA , Carhuapoma JR , et al. Guidelines for the management of aneurysmal subarachnoid hemorrhage: a guideline for healthcare professionals from the American Heart Association/American Stroke Association. Stroke 2012;43:1711–1737. 22556195 10.1161/STR.0b013e3182587839

[5] Rose JC , Mayer SA . Optimizing blood pressure in neurological emergencies. Neurocrit Care 2004;1:287–299. 16174926 10.1385/NCC:1:3:287

[6] Rajkomar A , Oren E , Chen K , et al. Scalable and accurate deep learning with electronic health records. NPJ Digital Med 2018;1:18. 10.1038/s41746-018-0029-1 PMC6550175 31304302

[7] Tabak YP , Sun X , Nunez CM , Johannes RS . Using electronic health record data to develop inpatient mortality predictive model: Acute Laboratory Risk of Mortality Score (ALaRMS). J Am Med Inform Assoc 2013;21:455–463. 24097807 10.1136/amiajnl-2013-001790 PMC3994855

[8] Shadmi E , Flaks‐Manov N , Hoshen M , et al. Predicting 30‐day readmissions with preadmission electronic health record data. Med Care 2015;53:283–289. 25634089 10.1097/MLR.0000000000000315

[9] Eapen ZJ , Liang L , Fonarow GC , et al. Validated, electronic health record deployable prediction models for assessing patient risk of 30‐day rehospitalization and mortality in older heart failure patients. JACC: Heart Fail 2013;1:245–251. 24621877 10.1016/j.jchf.2013.01.008

[10] Stevens RD , Sharma K . Predictive modeling in aneurysmal subarachnoid hemorrhage. Crit Care Med 2016;44:1613–1614. 27428125 10.1097/CCM.0000000000001808

[11] Jaja BN , Saposnik G , Lingsma HF , et al. Development and validation of outcome prediction models for aneurysmal subarachnoid haemorrhage: the SAHIT multinational cohort study. BMJ 2018;360:j5745. 29348138 10.1136/bmj.j5745

[12] Luo W , Phung D , Tran T , et al. Guidelines for developing and reporting machine learning predictive models in biomedical research: a multidisciplinary view. J Med Internet Res 2016;18:e323. 27986644 10.2196/jmir.5870 PMC5238707

[13] Stekhoven DJ , Bühlmann P . MissForest—non‐parametric missing value imputation for mixed‐type data. Bioinformatics 2011;28:112–118. 22039212 10.1093/bioinformatics/btr597

[14] Kuhn M . The caret package. Vienna, Austria: R Foundation for Statistical Computing https://cranr‐projectorg/package=caret. 2012.

[15] Caplan JM , Colby GP , Coon AL , et al. Managing subarachnoid hemorrhage in the neurocritical care unit. Neurosurg Clinic 2013;24:321–337. 10.1016/j.nec.2013.02.008 23809028

[16] Pickard J , Murray G , Illingworth R , et al. Effect of oral nimodipine on cerebral infarction and outcome after subarachnoid haemorrhage: British aneurysm nimodipine trial. BMJ 1989;298:636–642. 2496789 10.1136/bmj.298.6674.636 PMC1835889

[17] Abd‐Elsayed AA , Wehby AS , Farag E . Anesthetic management of patients with intracranial aneurysms. Ochsner J 2014;14:418–425. 25249809 PMC4171801

[18] Investigators N‐SS . Hypoglycemia and risk of death in critically ill patients. N Engl J Med 2012;367:1108–1118. 22992074 10.1056/NEJMoa1204942

[19] Frontera JA , Fernandez A , Claassen J , et al. Hyperglycemia after SAH: predictors, associated complications, and impact on outcome. Stroke 2006;37:199–203. 16339481 10.1161/01.STR.0000194960.73883.0f

[20] Williams G , Maroufy V , Rasmy L , et al. Vasopressor treatment and mortality following nontraumatic subarachnoid hemorrhage: a nationwide electronic health record analysis. Neurosurg Focus 2020;48:E4. 10.3171/2020.2.FOCUS191002 32357322

[21] Elliott R‐JS , Godoy DA , Michalek JE , et al. The effect of morbid obesity on subarachnoid hemorrhage prognosis in the United States. World Neurosurg 2017;105:732–736. 28642182 10.1016/j.wneu.2017.06.068

