# Supplementary material for: Machine learning prediction of the adverse outcome for nontraumatic subarachnoid hemorrhage patients
Source: Ann Clin Transl Neurol. 2020 Sep 29;7(11):2178–85. doi: 10.1002/acn3.51208 (PMC7664270; doi:10.1002/acn3.51208)
Supplement: Supplementary file 1 — Table S1. Missing rate summary. Table S2. Model discrimination summary. Table S3. Model calibration summary. Table S4. Auto machine learning implement comparison. Figure S1. Important variables for prediction with first 48 h EHR data. Figure S2. Important variables for prediction with first 48 h EHR data. Figure S3. Important variables for prediction after the next 24 h. Figure S4. Important variables for prediction after the next 48 h. Figure S5. Important variables for prediction after the next 72 h. Figure S6. Variables commonly included with first 24, 48, 72 h EHR data. Figure S7. Variables commonly included for the prediction after the next 24, 48, 72 h. [file ACN3-7-2178-s001.docx]

**SUPPLEMENTAL MATERIAL**

**Machine Learning Prediction of the Adverse Outcome for Nontraumatic Subarachnoid Hemorrhage Patients**

supplemental Pre-processing and Preparation

supplemental Prediction Methods

Supplemental Machine Learning Methods

supplemental Tables

Table S1. Missing rate summary

Table S2. Model discrimination summary

Table S3. Model calibration summary

Table S4. Auto machine learning implement comparison

**Supplemental Figures**

Figure S1. Important variables for prediction with first 48 hours EHR data

Figure S2. Important variables for prediction with first 72 hours EHR data

Figure S3. Important variables for prediction after the next 24 hours

Figure S4. Important variables for prediction after the next 48 hours

Figure S5. Important variables for prediction after the next 72 hours

Figure S6. Variables commonly included with first 24, 48, 72 hours’ EHR data

Figure S7. Variables commonly included for the prediction after the next 24, 48, 72 hours

**Supplemental References**

**supplemental Pre-processing and Preparation**

**Define the outcome**

With the Cerner database, the death status is indicated by the patient discharge disposition given an encounter. There are 24 valid (not labeled as none or not mapped) categories of patient discharge disposition in the cohort of SAH patients who have assigned at least one of three vasopressors (phenylephrine, norepinephrine, and dopamine). In this study, we consider patients who have one of the five categories of discharge disposition description in the following figure as adverse outcome cases. Subjects that had any other discharge disposition were assumed to be alive and not imminently about to die at discharge.

We are primarily interested in the association of initial choice of vasopressors and in-hospital mortality. We labeled each patient with one of the four groups based on the initial vasopressor treatments, i.e., norepinephrine only, phenylephrine only, dopamine only, and multiple initial vasopressors. A total of 40 patients who have multiple initial vasopressors were identified. Those patients were excluded in further data analysis.

**Encounter combination**

Initially, a total of 4850 SAH patients (including traumatic and nontraumatic SAH patients) were extracted from the Cerner database, with 4881 encounters. In the Cerner database, the time intervals of some encounters are overlapped, nested and closely related. We combined the encounters based on ‘24 hours’ rule: combine encounters with time gap (between next encounter beginning time and previous encounter end time) less than 24 hours. The combined encounters were considered as one visit. The ‘24 hours’ rule dropped a total of 17 encounters; the number of encounters that are kept for further data analysis is 4864.

**Cohort refining**

The inclusion and exclusion criteria initially defined the study cohort, but they are not enough to make sure all the patients in the initial cohort are eligible for data analysis. During the data processing and preparation step, we identified some patients that were not eligible for further data analysis. In this study, we excluded 40 patients because they had multiple initial assigned vasopressors. Patients who have missing outcome and gender were also excluded. Some patients were dropped from the study due to the error of age. For example, two patients were adults indicated by their age in year in the Cerner database. However, their diagnosis records showed they were pediatrics. More importantly, we excluded those patients who are traumatic SAH due to the distinct clinical characteristics between traumatic and nontraumatic SAH. The cohort refining procedures in this study is shown in the following figure. The cohort is originally extracted from the Cerner database with 4850 patients. After six different data preparation steps, a total of 2383 patients are excluded and 2467 nontraumatic SAH patients are included for the final data analysis.

**Data preparation for prediction scenarios**

Let $R_{t}$ denote as the time of records that used for prediction, $V_{t}$ is the initial vasopressor assigned time, $T_{0}$ is the SAH visit beginning time, $T_{e}$ is the SAH visit end time, thresholds are 24, 48, and 72 hours. In Scenario 1, only the EHR data that satisfy the following three conditions were included for prediction. Patients that have $T_{e}- T_{0} \leq threshold$ or $V_{t}> T_{0}+threshold$ were excluded for prediction.

- ${T_{0}\leq R}_{t}\leq T_{0}+threshold$
- ${T_{0}\leq V}_{t}\leq T_{0}+threshold$
- $T_{e}- T_{0} >threshold$

In Scenario 2, there require the following two conditions for the EHR data in the observational window of the prediction. Patients that have $T_{e}- T_{0} \leq threshold$ or $V_{t}> T_{e}-threshold$ were excluded for prediction. ${T_{0}\leq R}_{t}\leq T_{e}-threshold$

- ${T_{0}\leq V}_{t}\leq T_{e}-threshold$

Because we specifically predicted the risk of mortality for nontraumatic SAH patients who were treated with vasopressors, all the subjects in the two scenarios were required to have vasopressors in their observational window.

**supplemental Prediction Methods**

**Variables used in the model**

Age of the patient was categorized as <40, 40-49, 50-59, 60-69, and >70 years, consistent with the PHASES study.^1^ We considered three commonly used vasopressors, dopamine, norepinephrine, and phenylephrine. Thus, the first vasopressor treatment was a categorical variable with three levels. The baseline diagnosis, namely the diseases present on admission and chronic diseases^2^, were considered as binary variables. In the Cerner database, medications, procedures, lab tests, vital signs, and clinical observations have time-stamps, which allowed us to test different temporal prediction scenarios (see more detail in predicting setting and modeling below). For medications, we used generic names and only considered the first administration of that class of medications. Therefore, medication variables were treated as binary predictors. Medical procedures were treated as categorical variables with three levels (1) a procedure performed, (2) a procedure not performed, and (3) none of any procedures performed for this patient (visit). Lab tests, vital signs, and clinical observations had many missing values partially because not all patients were tested or measured for the same specific labs and clinical observations. Labs with repeated measures were represented using mean values. We then imputed the missingness of results from lab tests, vital signs, and clinical observations with MisForest.^3, 4^ We only considered variables that had an adequate number of outcome events per predictor variable (EPV), specifically, we required EVP$\geq$ 10 to consider including the variable in the model.^5^

**Leakage detection**

We excluded variables that could serve as surrogates for the mortality based on clinical judgment and exploratory data analysis. For example, we excluded diagnoses such as brain death (ICD-9-CM, 348.82) and encounter for palliative care (ICD-9-CM, V66.7). Diagnoses that are potential consequences of death were also excluded, e.g., donors of other specified organ or tissue (ICD-9-CM, V59.8), donors of unspecified organ or tissue (ICD-9-CM, V59.9), liver donor (ICD-9-CM, V59.6), and cornea donors (ICD-9-CM, V59.5). Variables that introduced the information of the length of stay were excluded from model development, such as continuous invasive mechanical ventilation for less than 96 consecutive hours, and continuous invasive mechanical ventilation for 96 consecutive hours or more.

**Model validation measures**

We used both discrimination and calibration metrics to validate the prediction models on the testing data set. In terms of model discrimination, the primary evaluation criterion was the AUC and corresponding 95% CI. We also reported other evaluation criteria such as sensitivity, specificity, positive predictive value, and negative predictive value in the supplementary materials. We systematically examined the goodness-of-fit of the model using the Hosmer-Lemeshow test.

**supplemental Machine Learning Methods**

To predict the risk of mortality, we explored six machine learning methods, including the elastic net regularized logistic regression approach, support vector machine (SVM), random forest, gradient boosting machine (GBM) and XGBoost, multilayer perceptron (MLP). Due to its property of interpretability and prediction accuracy, we mainly reported the results from the Elastic Net method (implemented with R package caret^6^) in the main text. Our results were further validated and confirmed from the aforementioned machine learning approaches using different auto-machine learning software platforms, including TPOT,^7^ H2O,^8^ and Auto-Sklearn.^9^  Here, we briefly reviewed each machine learning model.

**Elastic net regularized logistic regression**

Logistic regression is the most popular model for binary outcome data. It’s a special case of the Generalized Linear Model (GLM). A simple logistic regression model with one explanatory variable $X$can be written as

$logit\left[ \pi\left( x \right) \right]=\log\left( \frac{\pi\left( x \right)}{1-\pi\left( x \right)} \right)=$ $\beta_{0}+\beta x$

Where $\pi\left( x \right)=P\left( Y=1 | X=x \right)=1-P\left( Y=0 | X=x \right)$. The $i$th patient outcome mortality in this study can be denoted as $Y_{i}$, where $Y_{i}\in\{0, 1\}$. The estimates from a regular logistic regression are defined by

$$\hat{\beta}=arg\min_{\beta} \sum_{i=1}^{n} \left\{ y_{i}\ln\left[ \pi\left( x_{i} \right) \right]+\left( 1-y_{i} \right)\ln[1-\pi\left( x_{i} \right)] \right\}$$

The parameter $\beta$ can be interpreted as the change of the log-odds ratio of death when as one unit change in the value of $X$. When the number of variables is large, certain variable selection methods are needed to make the final model interpretable and even reduce the prediction error. Regularization or shrinkage methods combine the variable selection procedures with model building in a continuous way by adding penalties to the loss functions. The estimates from the elastic net regularized logistic regression are defined by

$$\hat{\beta}=arg\min_{\beta} \sum_{i=1}^{n} \left\{ y_{i}\ln\left[ \pi\left( x_{i} \right) \right]+\left( 1-y_{i} \right)\ln[1-\pi\left( x_{i} \right)] \right\}+\lambda_{1}\left\| \beta\right\|_{1}+\lambda_{2}\left\| \beta\right\|^{2}$$

where $\lambda_{1}\left\| \beta\right\|_{1}+\lambda_{2}\left\| \beta\right\|^{2}$ denotes the elastic net penalty.

**Support vector machine (SVM)**

One of the most influential approaches to supervised learning is the support vector machine^10, 11^. Compared to logistic regression, SVM aims to find a separating hyperplane that maximizes the distance of the closest points to the margin, instead of assuming a probabilistic model**.** Assuming training data consists of N pairs $\left( x_{1},y_{1} \right), \left( x_{2},y_{2} \right), \cdots\left( x_{N},y_{N} \right),$with $x_{i}\in R^{p}$ and $y_{i}$ ∈ {-1, 1}. Define a hyperplane by

{$x:f\left( x \right)=\beta_{0}+{h(x)}^{T}\beta$ = 0}

where $h(x)$ denotes the transformed feature vector. The inner product of $h(x)$, $K\left( x,x^{'} \right)= \left\langle h\left( x \right), h(x^{'}) \right\rangle$, could be replaced by varying kernel functions, such as follows.

dth-Degree polynomial: $K\left( x,x^{'} \right)={(1+\left\langle x, x^{'} \right\rangle)}^{d}$,

Radial basis: $K\left( x,x^{'} \right)={exp(-\gamma\left\| x-x^{'} \right\|}^{2})$,

Neural network: $K\left( x,x^{'} \right)=tanh(\kappa_{1}\left\langle x, x^{'} \right\rangle+\kappa_{2})$.

The SVM model solves the optimization problem

$$\min_{\beta_{0},\beta} \sum_{i=1}^{N} {[1-y_{i}f(x_{i})]}_{+}+\frac{\lambda}{2}\left\| \beta\right\|^{2}$$

where the subscript “+” indicates positive part.

**Random forest**

Random forest is a tree-based regression and classification method. Each tree is built on bootstrapped training samples and a weighted average of the trees is used for prediction. Only a random subset of variables is considered as a split candidate, which results in the majority of the available predictors being excluded from each tree building^12^.

**Gradient boosting machine (GBM) and XGboost**

Boosting is also a weighted averaging method which sequentially modifies the coefficients of regression or classification algorithm, thereby generating a sequence of models $G_{m}\left( x \right), m=1,2,\ldots, M.$ The prediction of a two-class problem, with the outcome labeled as $Y\in\{-1,1\}$, is obtained by weighted average vote:

$$G\left( x \right)=sign(\sum_{m=1}^{M} \alpha_{m}G_{m}(x))$$

where $\alpha_{1}, \alpha_{2},\ldots\alpha_{M}$ denotes the weight of the contribution from each respective $G_{m}(x)$, $G_{m}(x)\in\{-1,1\}$. In general, the classifier is replaced by the basis function expansions which takes the form

$$f\left( x \right)= \sum_{m=1}^{M} \beta_{m}b(x;\gamma_{m})$$

where $\beta_{m}, m=1,2,\ldots, M$ are the expansion coefficients, and $b(x;\gamma_{m})$ is usually some simple function of the $x$. A general boosting algorithm uses forward stagewise procedures to build the model^13^. The gradient boosting machine builds an additive tree model which is proposed by Friedman^14^. The numerical optimization connects the steepest descent rather and forwards stagewise procedure. The algorithm can be found in the book written by Hastie, Tibshirani and Friedman.^15^ XGboost is an efficient implementation of gradient boosting machines.^16^

**Multilayer perceptron (MLP)**

Multilayer perceptron (MLP), also known as artificial neural networks (ANNs), is one of the basic classifiers in deep learning. An ANNs with a single hidden layer can be written as:

$$h_{ij}= \sigma_{1}\left( W_{j}^{'}X_{i}+b_{j} \right)$$

$$P\left( Y_{i}=1 \right)= \sigma_{2}(W_{0}^{'}H_{i}+ b_{0})$$

where $W_{j}$is a p $\times$1weight vector, $X_{i}$is the i-th observation, $b_{j}$ is a scalar, j = 1, 2, $\cdots$, m. $H_{i}$ is the single hidden layer for i-th observation, $H_{i}$ = ($h_{i1,}h_{i2}, \cdots h_{im}$)'. $W_{0}$ is a m $\times$1weight vector, $b_{0}$ is a scalar. $\sigma_{1}$and $\sigma_{2} \mathrm{are}$ the active function, such as logistic sigmoid function, tanh function. $P\left( Y_{i}=1 \right)$ denotes the probability of outcome being present for the $i$th sample.

**supplemental Prediction Results**

Besides the AUC reported in the main text, other model evaluation criteria, such as sensitivity, specificity, positive predictive value, and negative predictive value (Table S1). To check the goodness of fit of the elastic net penalized logistic regression model, we performed Hosmer-Lemeshow test with df =48 (Table S2). All the p-values were larger than 0.05, we concluded that the predictive models fit the data well. In the main text, we mainly report the prediction results from the Elastic Net penalized logistic regression model (implemented with R package caret). The prediction results of the other machine learning approaches using different auto-machine learning software platforms, including TPOT, H2O, and Auto-Sklearn (Table S3).

**Supplemental Tables**

Table S1.Missing rate summary of lab and clinical observational variables (- denotes the variables are not included in the prediction cases).

| Variable | With first 24hours | With  first  48 hours | With first  72 hours | After next  24 hours | After next 48 hours | After next  72 hours |
| --- | --- | --- | --- | --- | --- | --- |
| base excess | 0.63 | 0.57 | 0.54 | 0.5 | 0.49 | 0.78 |
| blood pressure diastolic invasive | 0.78 | 0.79 | 0.87 | 0.8 | 0.79 | 0.79 |
| blood pressure systolic invasive | 0.78 | 0.78 | 0.87 | 0.79 | 0.79 | 0.79 |
| bmi | 0.7 | 0.7 | 0.85 | 0.67 | 0.66 | 0.65 |
| braden scale for predicting pressure ulcer risk | 0.68 | 0.68 | 0.68 | 0.68 | 0.68 | 0.83 |
| bsa | 0.85 | 0.86 | 0.92 | 0.82 | 0.81 | 0.81 |
| co2 total arterial | 0.55 | 0.54 | 0.55 | 0.55 | 0.55 | 0.77 |
| diastolic | 0.56 | 0.59 | 0.77 | 0.58 | 0.57 | 0.57 |
| fio2 fraction of inspired oxygen. | 0.64 | 0.66 | 0.67 | 0.66 | 0.67 | 0.83 |
| glasgow coma score | 0.59 | 0.6 | 0.6 | 0.59 | 0.59 | 0.79 |
| hco3 arterial | 0.4 | 0.35 | 0.32 | 0.29 | 0.28 | 0.65 |
| heart rate | 0.58 | 0.6 | 0.78 | 0.59 | 0.58 | 0.58 |
| height | 0.66 | 0.67 | 0.83 | 0.63 | 0.62 | 0.62 |
| mean arterial pressure | 0.61 | 0.63 | 0.79 | 0.62 | 0.61 | 0.61 |
| o2 saturation arterial sao2. | 0.51 | 0.49 | 0.47 | 0.45 | 0.45 | 0.73 |
| pain scale score | 0.84 | 0.82 | 0.82 | 0.79 | 0.79 | - |
| pco2 arterial | 0.44 | 0.41 | 0.41 | 0.39 | 0.39 | 0.69 |
| peep positive end expiratory pressure. | 0.75 | 0.75 | 0.75 | 0.75 | 0.75 | 0.87 |
| Ph arterial | 0.47 | 0.43 | 0.42 | 0.41 | 0.4 | 0.7 |
| pip peak inspirator pressure. | 0.78 | 0.8 | 0.8 | 0.8 | 0.8 | - |
| po2 arterial | 0.38 | 0.34 | 0.32 | 0.28 | 0.28 | 0.66 |
| pulse rate | 0.76 | 0.78 | 0.88 | 0.73 | 0.73 | 0.73 |
| respiratory rate | 0.56 | 0.59 | 0.77 | 0.58 | 0.57 | 0.57 |
| spo2 saturation of peripheral oxygen. | 0.8 | 0.82 | 0.82 | 0.82 | 0.82 | - |
| systolic | 0.56 | 0.59 | 0.77 | 0.58 | 0.57 | 0.57 |
| temperature | 0.58 | 0.6 | 0.79 | 0.58 | 0.58 | 0.58 |
| tidal volume | 0.82 | 0.83 | 0.83 | 0.82 | 0.82 | - |
| weight | 0.6 | 0.63 | 0.8 | 0.59 | 0.59 | 0.59 |
| partial thromboplastin time | 0.36 | 0.35 | 0.33 | 0.33 | 0.32 | 0.31 |
| creatinine,serum quantitative | 0.05 | 0.02 | 0.02 | 0.02 | 0.02 | 0.02 |
| specific gravity, urine | 0.73 | 0.71 | 0.67 | 0.47 | 0.46 | 0.45 |
| anion gap | 0.35 | 0.34 | 0.32 | 0.29 | 0.28 | 0.28 |
| basophils percent | 0.54 | 0.5 | 0.48 | 0.39 | 0.38 | 0.37 |
| basophils count | 0.62 | 0.6 | 0.58 | 0.53 | 0.51 | 0.51 |
| blood gas pco2, arterial | 0.41 | 0.4 | 0.4 | 0.4 | 0.41 | 0.41 |
| blood gas ph, arterial | 0.4 | 0.38 | 0.38 | 0.4 | 0.39 | 0.39 |
| blood gas po2, arterial | 0.46 | 0.47 | 0.47 | 0.48 | 0.48 | 0.48 |
| eosinophil percent | 0.66 | 0.65 | 0.62 | 0.54 | 0.54 | 0.54 |
| inr (international normalized ratio) | 0.39 | 0.35 | 0.35 | 0.34 | 0.34 | 0.33 |
| mean corpuscular hemoglobin | 0.19 | 0.16 | 0.15 | 0.15 | 0.15 | 0.15 |
| mean corpuscular hemoglobin concentration | 0.07 | 0.04 | 0.03 | 0.03 | 0.02 | 0.03 |
| mean corpuscular volume | 0.06 | 0.03 | 0.03 | 0.02 | 0.02 | 0.02 |
| mean platelet volume | 0.21 | 0.14 | 0.13 | 0.13 | 0.12 | 0.12 |
| monocyte percent | 0.57 | 0.54 | 0.52 | 0.45 | 0.45 | 0.45 |
| neutrophil | 0.64 | 0.6 | 0.58 | 0.53 | 0.51 | 0.51 |
| ph, urine | 0.72 | 0.71 | 0.69 | 0.5 | 0.49 | 0.48 |
| red blood cell distribution width (rdw) | 0.26 | 0.23 | 0.21 | 0.21 | 0.2 | 0.2 |
| prothrombin time | 0.28 | 0.28 | 0.27 | 0.27 | 0.26 | 0.26 |
| potassium, serum | 0.05 | 0.02 | 0.02 | 0.01 | 0.01 | 0.01 |
| magnesium | 0.54 | 0.47 | 0.42 | 0.31 | 0.3 | 0.3 |
| albumin, serum | 0.51 | 0.48 | 0.46 | 0.37 | 0.37 | 0.37 |
| alkaline phosphatase, serum | 0.5 | 0.5 | 0.5 | 0.4 | 0.4 | 0.4 |
| blood urea nitrogen | 0.05 | 0.02 | 0.02 | 0.02 | 0.02 | 0.02 |
| calcium, serum | 0.24 | 0.22 | 0.21 | 0.2 | 0.2 | 0.19 |
| carbon dioxide co2 | 0.46 | 0.47 | 0.47 | 0.47 | 0.47 | 0.47 |
| chloride, serum | 0.09 | 0.05 | 0.05 | 0.05 | 0.05 | 0.05 |
| hemoglobin | 0.05 | 0.03 | 0.02 | 0.02 | 0.02 | 0.02 |
| ck/cpk, total, serum | 0.67 | 0.67 | 0.67 | 0.61 | 0.62 | 0.62 |
| hematocrit | 0.06 | 0.03 | 0.02 | 0.03 | 0.02 | 0.02 |
| white blood cell count | 0.06 | 0.03 | 0.03 | 0.03 | 0.02 | 0.02 |
| red blood cell count | 0.28 | 0.27 | 0.26 | 0.24 | 0.24 | 0.24 |
| sodium, serum | 0.08 | 0.04 | 0.05 | 0.04 | 0.04 | 0.03 |
| bicarbonate hco3 | 0.75 | 0.72 | 0.71 | 0.7 | 0.69 | 0.69 |
| glucose, serum/plasma quantitative | 0.2 | 0.14 | 0.12 | 0.12 | 0.11 | 0.11 |
| osmolality, serum | 0.85 | 0.77 | 0.73 | 0.64 | 0.62 | 0.62 |
| phosphorus, serum | 0.69 | 0.56 | 0.48 | 0.35 | 0.34 | 0.34 |
| platelet count | 0.18 | 0.12 | 0.11 | 0.12 | 0.11 | 0.11 |
| protein total, serum | 0.46 | 0.47 | 0.47 | 0.38 | 0.38 | 0.38 |
| alanine aminotransferase / sgpt | 0.48 | 0.47 | 0.47 | 0.37 | 0.37 | 0.37 |
| bilirubin total serum or plasma mass/volume | 0.52 | 0.52 | 0.51 | 0.42 | 0.42 | 0.42 |
| aspartate aminotransferase / sgot | 0.46 | 0.45 | 0.44 | 0.33 | 0.33 | 0.33 |
| eosinophil count | 0.56 | 0.51 | 0.49 | 0.41 | 0.39 | 0.39 |
| weight..ideal | - | 0.89 | 0.95 | 0.85 | 0.84 | 0.84 |
| ua white blood cell | - | 0.86 | 0.84 | 0.75 | 0.74 | 0.73 |
| creatine kinase-mb | - | 0.84 | - | 0.83 | 0.83 | 0.84 |
| etco2..end.tidal.co2. | - | - | 0.87 | 0.86 | 0.86 | - |
| fibrinogen | - | - | 0.89 | 0.85 | 0.85 | 0.85 |
| cerebral.perfusion.pressure..cpp. | - | - | - | 0.88 | 0.88 | 0.87 |
| pco2..capillary | - | - | - | 0.88 | 0.88 | - |
| ph..capillary | - | - | - | 0.88 | 0.88 | - |
| osmolality, urine | - | - | - | 0.8 | 0.79 | 0.79 |
| sodium, urine | - | - | - | 0.85 | 0.84 | 0.83 |

Table S2. Model discrimination summary with optimal cutoff point.

| Measure | Predict with first 24hours | Predict with first 48hours | Predict with first 72hours | Predict after next 24hours | Predict after next 48hours | Predict after next 72hours |
| --- | --- | --- | --- | --- | --- | --- |
| Optimal J | 0.50 | 0.71 | 0.66 | 0.80 | 0.73 | 0.83 |
| Sensitivity | 0.85 | 0.95 | 0.76 | 0.91 | 0.87 | 0.88 |
| Specificity | 0.77 | 0.62 | 0.81 | 0.82 | 0.87 | 0.81 |
| NPV | 0.87 | 0.96 | 0.89 | 0.96 | 0.95 | 0.96 |
| PPV | 0.75 | 0.59 | 0.62 | 0.68 | 0.69 | 0.54 |
| Accuracy | 0.81 | 0.74 | 0.79 | 0.85 | 0.87 | 0.82 |

Optimal J: the optimal cutoff point by maximizing Youden Index, which is defined as $J= {max}_{i}({sensitivity}_{i}+{specificity}_{i}-1)$.^17^

NPV: negative predictive value.

PPV: positive predictive value.

Table S3. Model calibration summary based on Hosmer-Lemeshow test, df = 48.

|  | Predict with first 24hours | Predict with first 48hours | Predict with first 72hours | Predict after next 24hours | Predict after next 48hours | Predict after next 72hours |
| --- | --- | --- | --- | --- | --- | --- |
| $\chi^{2}$ test statistics | 53.39 | 57.15 | 51.47 | 28.82 | 37.62 | 50.03 |
| p-value | 0.27 | 0.17 | 0.34 | 0.99 | 0.86 | 0.39 |

Table S4. The AUC of the risk of mortality based on different models and auto machine learning (Auto-ML) platforms. -- denotes the machine learning model is not available in the Auto-ML platform.

| Prediction cases | Auto-ML  AUC | caret | tpot | h2o | auto-sklearn |
| --- | --- | --- | --- | --- | --- |
| Risk of death after the next 24 hours | SVM  model | 0.94 | 0.94 | -- | 0.94 |
|  | Random forest | 0.92 | 0.91 | 0.91 | 0.92 |
|  | Penalized logistic | 0.93 | 0.93 | 0.93 | 0.94 |
|  | GBM | 0.93 | -- | 0.93 | 0.92 |
|  | MLP | 0.92 | -- | 0.94 | -- |
|  | XGboost | -- | 0.92 | 0.93 | 0.93 |
| Risk of death after the next 48 hours | SVM | 0.92 | 0.93 |  | 0.94 |
|  | Random forest | 0.91 | 0.91 | 0.91 | 0.92 |
|  | Penalized logistic | 0.92 | 0.93 | 0.92 | 0.93 |
|  | GBM | 0.93 | -- | 0.93 | 0.92 |
|  | MLP | 0.92 | -- | 0.94 | -- |
|  | XGboost | -- | 0.92 | 0.93 | 0.93 |
| Risk of death after the next 72 hours | SVM | 0.92 | 0.9 | -- | 0.92 |
|  | Random forest | 0.84 | 0.87 | 0.87 | 0.89 |
|  | Penalized logistic | 0.91 | 0.91 | 0.91 | 0.9 |
|  | GBM | 0.88 | -- | 0.89 | 0.91 |
|  | MLP | 0.9 | -- | 0.91 | -- |
|  | XGboost | -- | 0.89 | 0.9 | 0.88 |
| Risk of death after the first 24 hours | SVM | 0.88 | 0.88 | -- | 0.88 |
|  | Random forest | 0.87 | 0.86 | 0.87 | 0.87 |
|  | Penalized logistic | 0.88 | 0.87 | 0.88 | 0.88 |
|  | GBM | 0.83 | -- | 0.86 | 0.87 |
|  | MLP | 0.83 | -- | 0.85 | -- |
|  | XGboost | -- | 0.88 | 0.85 | 0.87 |
| Risk of death after the first 48 hours | SVM | 0.85 | 0.85 | -- | 0.88 |
|  | Random forest | 0.87 | 0.84 | 0.86 | 0.86 |
|  | Penalized logistic | 0.87 | 0.85 | 0.87 | 0.87 |
|  | GBM | 0.83 | -- | 0.83 | 0.88 |
|  | MLP | 0.83 | -- | 0.83 | -- |
|  | XGboost | -- | 0.86 | 0.86 | 0.86 |
| Risk of death after the first 72 hours | SVM | 0.82 | 0.82 | -- | 0.88 |
|  | Random forest | 0.81 | 0.82 | 0.83 | 0.84 |
|  | Penalized logistic | 0.84 | 0.82 | 0.85 | 0.88 |
|  | GBM | 0.81 | -- | 0.84 | 0.82 |
|  | MLP | 0.75 | -- | 0.84 | -- |
|  | XGboost | -- | 0.84 | 0.84 | 0.83 |

**Supplemental Figures**

Figure S1. The predictors that were included in the model for predicting morality with the first 48 hours’ EHR data, and corresponding odds ratios in Scenario 1.

Figure S2. The predictors that were included in the model for predicting morality with the first 72 hours’ EHR data, and corresponding odds ratios in Scenario 1.

Figure S3. Important variables for predicting mortality after the next 24 hours with odds ratios greater than 1.05 or less than 0.95 in Scenario 2.

Figure S4. Important variables for predicting morality after the next 48 hours with odds ratios greater than 1.05 or less than 0.95 in Scenario 2.

Figure S5. Important variables for predicting morality after the next 72 hours with odds ratios greater than 1.05 or less than 0.95 in Scenario 2.

Figure S6. The range of odds ratio for predictors that were commonly included in the models for predicting mortality with only the first 24, 48 and 72 hours.

Figure S7. The range of odds ratios for predictors that were commonly included in the models for predicting mortality after the next 24, 48 and 72 hours.

**Supplemental References**

1. Greving JP, Wermer MJ, Brown Jr RD, et al. Development of the PHASES score for prediction of risk of rupture of intracranial aneurysms: a pooled analysis of six prospective cohort studies. *The Lancet Neurology* 2014; 13: 59-66.

2. Yoon J and Chow A. Comparing chronic condition rates using ICD-9 and ICD-10 in VA patients FY2014–2016. *BMC health services research* 2017; 17: 572.

3. Stekhoven DJ. missForest: Nonparametric missing value imputation using random forest. *Astrophysics Source Code Library* 2015.

4. Stekhoven DJ and Bühlmann P. MissForest—non-parametric missing value imputation for mixed-type data. *Bioinformatics* 2011; 28: 112-118.

5. Luo W, Phung D, Tran T, et al. Guidelines for developing and reporting machine learning predictive models in biomedical research: a multidisciplinary view. *Journal of medical Internet research* 2016; 18: e323.

6. Kuhn M. The caret package. *R Foundation for Statistical Computing, Vienna, Austria URL* [*https://cran*](https://cran) *r-project org/package= caret* 2012.

7. Olson RS, Bartley N, Urbanowicz RJ, et al. Evaluation of a tree-based pipeline optimization tool for automating data science. In: *Proceedings of the Genetic and Evolutionary Computation Conference 2016* 2016, pp.485-492. ACM.

8. Candel A, Parmar V, LeDell E, et al. Deep learning with H2O. *H2O ai Inc* 2016.

9. Feurer M, Klein A, Eggensperger K, et al. Efficient and robust automated machine learning. In: *Advances in neural information processing systems* 2015, pp.2962-2970.

10. Cortes C and Vapnik V. Support-vector networks. *Machine learning* 1995; 20: 273-297.

11. Boser BE, Guyon IM and Vapnik VN. A training algorithm for optimal margin classifiers. In: *Proceedings of the fifth annual workshop on Computational learning theory* 1992, pp.144-152.

12. Breiman L. Random forests. *Machine learning* 2001; 45: 5-32.

13. James G, Witten D, Hastie T, et al. *An introduction to statistical learning*. Springer, 2013.

14. Friedman JH. Greedy function approximation: a gradient boosting machine. *Annals of statistics* 2001: 1189-1232.

15. Friedman J, Hastie T and Tibshirani R. *The elements of statistical learning*. Springer series in statistics New York, 2001.

16. Chen T, He T, Benesty M, et al. Xgboost: extreme gradient boosting. *R package version 04-2* 2015: 1-4.

17. Youden WJ. Index for rating diagnostic tests. *Cancer* 1950; 3: 32-35.
